# A very large-scale microelectrode array for cellular-resolution electrophysiology

**DOI:** 10.1038/s41467-017-02009-x

**Published:** 2017-11-27

**Authors:** David Tsai, Daniel Sawyer, Adrian Bradd, Rafael Yuste, Kenneth L. Shepard

**Affiliations:** 1 Department of Electrical Engineering, Columbia University, New York, NY 10027 USA; 2 Departments of Biological Sciences and Neuroscience, Columbia University, New York, NY 10027 USA; 3 Departments of Electrical and Biomedical Engineering, Columbia University, New York, NY 10027 USA

## Abstract

In traditional electrophysiology, spatially inefficient electronics and the need for tissue-to-electrode proximity defy non-invasive interfaces at scales of more than a thousand low noise, simultaneously recording channels. Using compressed sensing concepts and silicon complementary metal-oxide-semiconductors (CMOS), we demonstrate a platform with 65,536 simultaneously recording and stimulating electrodes in which the per-electrode electronics consume an area of 25.5 μm by 25.5 μm. Application of this platform to mouse retinal studies is achieved with a high-performance processing pipeline with a 1 GB/s data rate. The platform records from 65,536 electrodes concurrently with a ~10 µV r.m.s. noise; senses spikes from more than 34,000 electrodes when recording across the entire retina; automatically sorts and classifies greater than 1700 neurons following visual stimulation; and stimulates individual neurons using any number of the 65,536 electrodes while observing spikes over the entire retina. The approaches developed here are applicable to other electrophysiological systems and electrode configurations.

## Abstract (web summary)

Large electronics limit low-noise, non-invasive electrophysiological measurements to a thousand simultaneously recording channels. Here the authors build an array of 65k simultaneously recording and stimulating electrodes and use it to sort and classify single neurons across the entire mouse retina.

## Introduction

The ability to observe and manipulate, with single-cell precision, the activities of large neuronal populations is an essential step toward better understanding of the nervous system^1^. It is now possible to observe activities in large neuronal populations through calcium imaging^2–4^. However, intracellular calcium concentration is influenced by many simultaneously active and mutually interacting mechanisms, including voltage- and ligand-gated calcium channels, intracellular stores, and chelation by proteins and by the fluorescent calcium indicator^5^. Many of these are not under direct control of the experimentalist and operate over a time scale substantially longer than that of action potentials, thus complicating interpretation. In particular, inferring action potentials from calcium signals require non-trivial calibration^6^, particularly if one is interested in precise spike timing and not just the number of spikes.

Extracellular electrophysiology, in contrast, enables direct read out of spikes. Recent progress has substantially increased the number of simultaneously recordable neurons^7^. However, the observable neurons, ranging from few tens to approximately a thousand^8–10^, remains small relative to the number of neurons in the brain. This is primarily due to trade-offs between several competing requirements: adequate recording signal-to-noise ratio (SNR), minimal biological invasiveness, at-scale recording, and, ideally, ability to stimulate the recorded neurons with precision.

Because of spatial requirements, having a complete amplifier chain and digitizer for each electrode, with sufficient noise performance, bandwidth and dynamic range, is infeasible for at-scale electrophysiology. Consequently, time-division multiplexing has been the mainstay for increasing density and channel count^9,11,12^, by sharing one signal path between several inputs. Traditional time-division multiplexing constitutes a sampled system. Since the input channels are sequentially scanned (sampled) over time, per-channel low-pass filters are required to reject frequencies above half the sampling rate (Supplementary Fig. 1a). Failure to do so causes aliasing of frequencies above half the per-channel sampling rate, thus degrading the SNR. Moreover, the more channels an inadequately filtered system has, the greater the SNR degradation.

The physical dimension of these filters dictates the density limit of large-scale electrophysiology. With an ideal noise ceiling of approximately 10 µV rms^13^, a parsimonious, multiplexed recorder, consisting of the electrode, an antialiasing filter and an amplifier, cannot be smaller than ~10,000 µm^2^ to ensure adequate noise performance, due to the capacitance density available in today’s microelectronic technologies (Supplementary Fig. 1b; also note Supplementary Fig. 1 caption). With mammalian neuronal soma ≤25 µm in diameter, this 20-to-1 dimensional mismatch fundamentally restricts the scalability of current approaches in electrophysiology. Due to this noise-verse-density trade-off, today’s large-scale neural recorders have either low noise but low simultaneously recording channel count^8,14^ or high channel count but high noise^11,15,16^.

Here we present a large-scale, high-density and low noise electrophysiological recording and stimulation platform based on CMOS electronics and an acquisition paradigm that negates the requirement for per-channel antialiasing filters, thereby overcoming scaling limitations faced by existing systems. This allows us to maintain 10 µV rms recording noise with per-channel electronics for 65,536 channels at a 25.5-µm electrode pitch, avoiding the common trade-off between density, channel count and noise^8,11,14–19^. We then demonstrate the platform’s ability to record from 10 s of thousands of neurons simultaneously. In conjunction with visual stimuli and the platform’s high-performance computing infrastructure, the system could functionally classify more than 1700 neurons in the retina automatically, including identification of rare cell types. Finally, combining cellular-resolution microstimulators with dense recordings across the entire retina allowed us to re-examine how electrical stimulation recruits neurons in a network, and importantly, how focal activation could be achieved.

## Results

### Compressed sensing-inspired electrophysiology

**Fig. 1:**
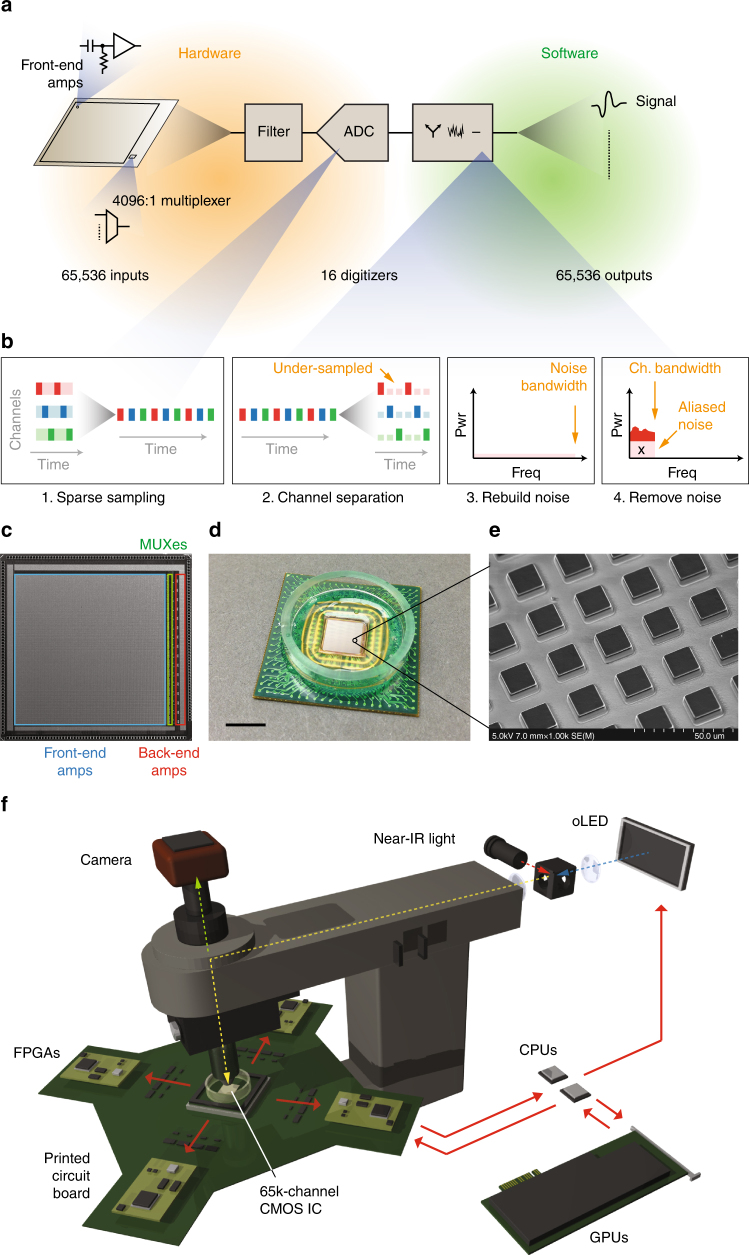
Platform for dense, large-scale electrophysiology. **a** Overview of acquisition paradigm based on compressed sensing. **b** Sparse sampling by the ADC, followed by digital reconstruction and removal of the spectral contribution by the aliased thermal noise. **c** A 65,536-channel recording and microstimulation grid based on CMOS-integrated circuits (IC). **d** Packaged IC. **e** Scanning electron micrograph of the 14 × 14 µm electrodes, spaced 25.5 µm apart. **f** Illustration of the experimental platform consisting of: the IC; supporting circuits; data processing pipeline containing CPUs and GPUs; and optics for near-infrared visualization and visual pattern delivery. ADC, analog-to-digital-converter; MUX, multiplexer

We constructed a 65,536-channel, multiplexed, extracellular electrophysiology system consisting of a recording and stimulation array based on a custom integrated circuit (IC), circuit-board-level analog and digital circuits and custom software libraries (Fig. 1a). Traditional implementations of such multiplexed systems incorporate per-channel low-pass filters to prevent noise aliasing (Supplementary Fig. 1a). Our approach (Fig. 1b, Supplementary Fig. 1b) does not use these filters, but instead, avoids aliasing using concepts from compressed sensing^20,21^. This is made possible by noting several characteristics of extracellular recordings and thermal noise aliasing.

First, electrophysiological recording is dominated by thermal noise at frequencies above a few kHz. It is a stationary process with a Gaussian time-domain amplitude distribution and uniform frequency distribution, up to the recording channel’s bandwidth. Therefore, this thermal noise can be described, and generated computationally, with only two parameters, its variance and bandwidth. Second, thermal noise aliasing offers two averaging properties, which greatly simplify the reconstruction, and subsequent removal, of its spectral contribution in the under-sampled, per-channel data. The power of thermal noise is approximately uniform. As the thermal noise powers are folded down into the first Nyquist zone (Supplementary Fig. 3b) during aliasing, the slight variations in power between frequencies are averaged out. This allows us to compute the power contributed by aliasing using the expected average thermal noise power, multiplied by the number of folded Nyquist zones. Similarly, the spectral angles of thermal noise have a uniform distribution with zero mean. The angle variation between frequencies converge to zero as the aliased thermal noise are folded down into the first Nyquist zone.

Taking advantage of the foregoing characteristics, we can digitally reconstruct the spectral contributions originating from the under-sampled thermal noise, then remove them from the sparsely-sampled channel data, thereby minimizing the effects of aliasing, without using per-channel anti-aliasing filters.

This acquisition strategy allows us to pack 65,536 channels (Fig. 1c, d) into an area of 42.6 mm^2^, with 25.5 µm spacing between channels (Fig. 1e), using CMOS IC processes. Each channel can be sampled at 10 kHz during full-grid recordings, with higher sampling rates achievable by reducing the recording area. Importantly, this platform does not have the noise-verses-density trade-off of classical large-scale electrophysiology. We constructed an electrophysiological platform based on this acquisition paradigm. It consists of the aforementioned 65,536-channel CMOS IC, custom circuit boards with filters and field programmable gate arrays (FPGAs), CPUs, graphical processing units (GPUs), and an OLED display for generating visual patterns (Fig. 1f).

### Achieving 65,536-channel recordings with minimal noise

**Fig. 2:**
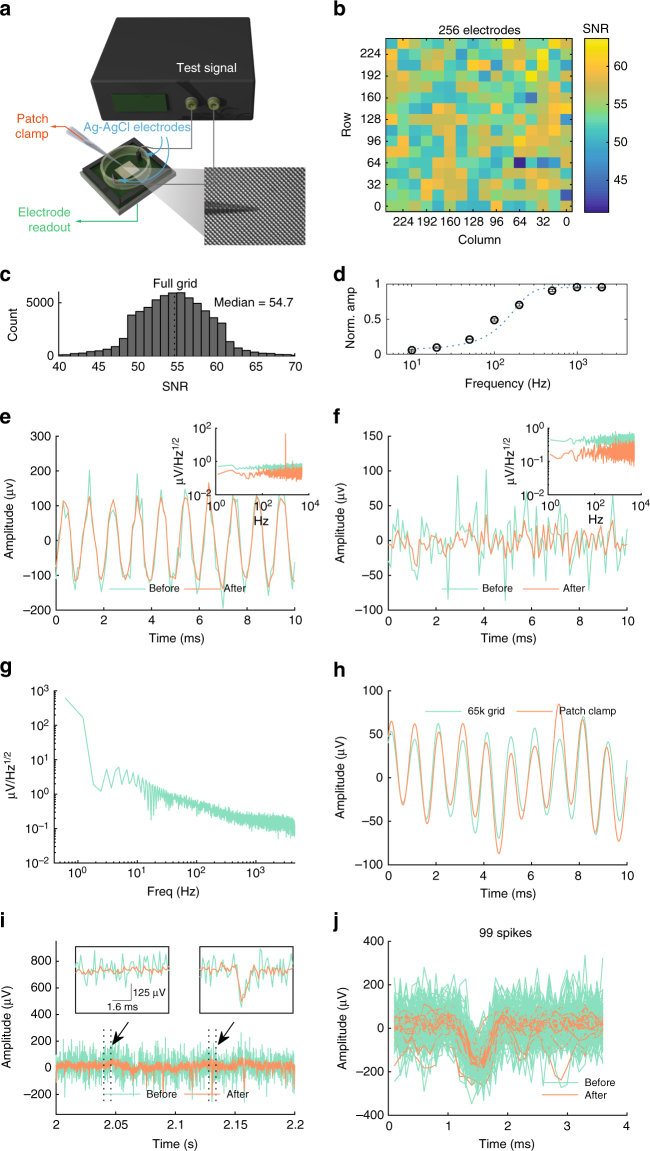
System performance characterization. **a** Test setup. Recordings from the 65,536-channel grid are compared against patch clamp recordings. Inset photo: pipette above the grid. **b** SNR variation across the array, measured at every sixteenth row and column. The test signal was a bath-applied 1-kHz, 200 µV sine wave. **c** SNR distribution for all electrodes in the array. **d** Frequency response of the capacitive recording front-end (mean ± SEM, 8 electrodes). **e**, **f** Comparison of per-channel data, before (green) and after (orange) removal of aliased thermal noise, for a recording with 1 kHz signal **e** and a baseline recording **f**. Insets in **e** and **f**: PSD plots of time-domain data in **e** and **f**, respectively. The dc component has been removed to better illustrate the linear reduction in noise floor across frequencies. **g** The system’s input referred noise was ~10 µV rms over 100–10k Hz, in saline. **h** Comparison to patch clamp amplifier recordings. The test signal was a 1 kHz, 100 µV peak-to-peak sine wave. Both traces have been bandpass filtered between 300–3k Hz for clarity. **i** Recordings before and after removing aliased thermal noise. Inset: expanded view of segment without and with spike, respectively. **j** Overlaid traces for 99 spike segments before and after removing aliased noise. Traces in **i** and **j** are unfiltered

To test the recording performance, we applied test signals through a pair silver-silver chloride electrodes into the recording chamber filled with physiological saline (Fig. 2a). The median SNR across the array was 54.9 with 200 µV test signals (Fig. 2b, c). Our system uses a capacitive recording interface^15,22,23^, formed by a 6-nm thick HfO_2_ dielectric deposited above each electrode and a pseudo-resistor constructed from a p-type MOSFET. The corner frequency is user-tuneable, and is nominally set to 100 Hz (Fig. 2d).

Each channel is typically observed at a rate well below the channel bandwidth. To minimize thermal noise aliasing, the spectral contribution of the under-sampled thermal noise is computed, then removed, from the channel data (Fig. 1b, steps 3–4). Figure 2e compares, for a 1 kHz signal applied in the saline-filled recording bath, the channel data before (green) and after (orange) removal of the aliased thermal noise. The SNR improvement is also apparent in the spectral domain (Fig. 2e inset). The noise floor was reduced uniformly across frequencies. Fig. 2f illustrates the effects of our processing strategy on a segment of baseline recording without test signal. Here, the noise was reduced from 21.7 µV rms (green) to 10.02 µV rms (orange) over the 100–3 kHz bandwidth following signal processing.

When recording in physiological saline, the 65k-electrode grid had ~10 µV rms input referred noise over the 100–5 kHz bandwidth, encompassing the spike frequency range of 300–3 kHz (Fig. 2g). Finally, the per-channel signal from the 65k-electrode grid closely resembled those from patch clamp recordings—the gold standard in electrophysiology–performed adjacent to the test electrode (Fig. 2h). For clarity, both traces were bandpass filtered between 300–3 kHz. The low-frequency fluctuation was due to noise pick-up by the wires connecting the signal generator to the bath electrodes.

To assess the performance of the de-noising procedure on biological recordings, Fig. 2i compares a recording before (green) and after (orange) removal of aliased noise. In particular, the inset plots in Fig. 2i show that the procedure reduced noise fluctuations without degrading the action potential waveforms. This is further illustrated in Fig. 2j for 99 spikes recorded from one electrode. The average variance of these raw waveforms is 8307.6. After removing the aliasing-induced spurious fluctuations, the average variance of the processed waveforms is 3179.0. While the amplitudes of signal and noise are both reduced, the SNR is improved by reducing the spectral contribution of aliased noise from the first Nyquist zone (Supplementary Fig. 3b verses Supplementary Fig. 3c).

Collectively, these results demonstrate the ability of this system to acquire, with high SNR, weak signals having amplitudes typical of mammalian extracellular recording, and to do so at spatial resolutions down to 25.5 µm, while simultaneously providing observable spatial coverage of 42.6 mm^2^ with 65,536 electrodes.

### Simultaneous recordings from more than 34,000 electrodes

**Fig. 3:**
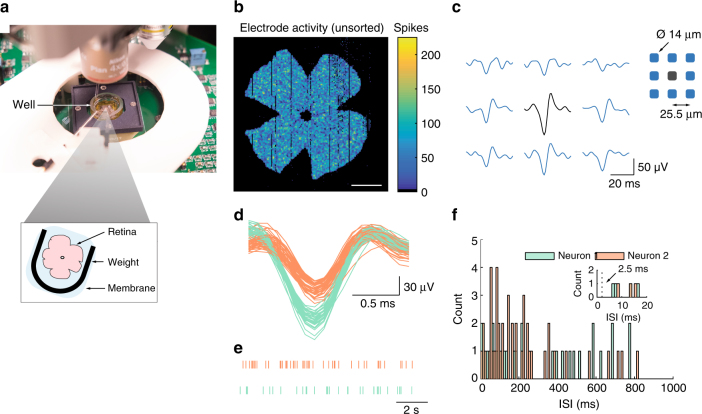
Large-scale recordings in the retina. **a** Photo of setup with mouse retina. **b** Spontaneous spiking activities recorded simultaneously from 34,187 electrodes over 12 s, at 10 kHz per electrode. Scale bar, 1275 µm. **c** Unit activities were observed concurrently on several adjacent electrodes spaced 25.5 µm apart. These spikes are single-trial waveforms. Sorted spike waveforms **d** and spike time raster **e** for one electrode in **b**. There are 33 and 47 spikes for the first and second neuron, respectively. **f** Inter-spike interval (ISI) plot for the two neurons in **d**. Inset: zoomed-in view of the first 20 ms

We tested the ability of the platform to carry out at-scale, cellular-resolution recordings with single-spike sensitivity by placing a piece of mouse retina, retinal ganglion cell (RGC) side down, on the recording grid (Fig. 3a). We began by observing the neurons’ spontaneous activities under scotopic conditions (Fig. 3b). Spikes were readily apparent, with 34,187 electrodes picking up spiking activities. This exceeded the best existing attempts at across-retina spike recordings by an order of magnitude in channel count^24^ and the best calcium imaging efforts in the retina by approximately two orders of magnitude^25,26^.

**Fig. 4:**
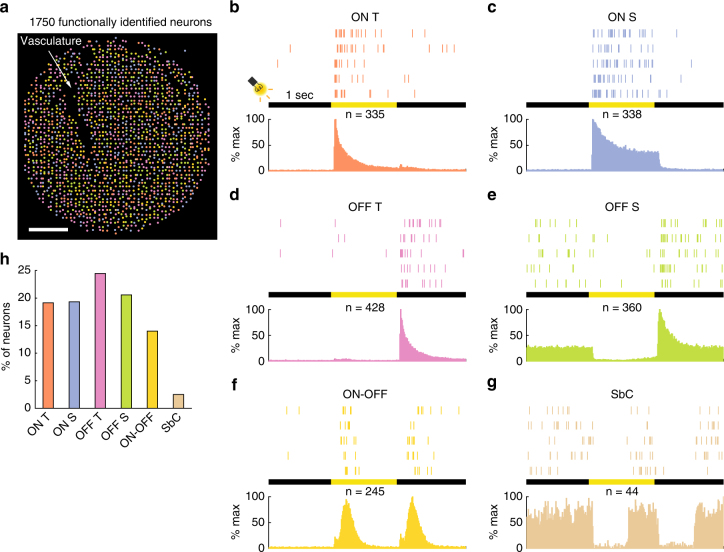
Functional classification of neurons using automated processing pipeline. The stimuli were 1-s, 1122-µm diameter, light spots. **a** Data from a sensing area of 1377 × 1275 µm were automatically analysed and, upon satisfying appropriate response characteristics, classified into one of six functional classes. Scale bar, 225 µm. **b**–**g** Raster plot for a representative ON transient RGC **b**, ON sustained RGC **c**, OFF transient RGC **d**, OFF sustained RGC **e**, ON–OFF RGC **f**, and suppression-by-contrast (SbC) RGC **g**, to five repetitions of one-second light flash. The peri-stimulus time histogram (10 ms bins) beneath each raster shows the normalized firing rate for all detected neurons in each class. The n-numbers denote the neurons considered in each histogram. All neurons were recorded simultaneously. **h** Distribution of functional classes for the identified neurons in **a**

Spikes from each neuron were observed on multiple adjacent electrodes (Fig. 3c, Supplementary Fig. 4e) and each electrode acquired spikes from more than one neuron (Fig. 3d, Supplementary Fig. 4c). Spike sorting accuracy is substantially improved by combining spatially dependent waveforms from adjacent electrodes^27^ (Supplementary Fig. 4), made possible by the dense 25.5 µm electrode pitch. Figure 3d, e illustrate the sorted spike waveforms and rasters, respectively, from one electrode in Fig. 3b. Note the lack of inter-spike intervals (ISIs) less than or equal to 2.5 ms (Fig. 3f), the typical absolute refractory period of mammalian neurons. The presence of such intervals would be indicative of incorrect clustering. A similar absence of ISI violation was also observed when we sorted electrodes with an order of magnitude more events (Supplementary Fig. 4a–d). The high electrode density also allowed us to triangulate the putative location of each observed neuron on the basis of small changes in waveform amplitude over space (Fig. 4a).

### Functional classification of neurons

There have been intense debates over the number of functional types of RGCs, the retina’s output neurons, and hence the number of information channels connecting the eyes to the brain. In the mouse, it has been variously estimated to be: >12 by traditional sparse electrophysiology^28^, ~12 types on morphological basis^29^, ≥16 by genetic markers^30^, and, most recently, ≥ 30 types with two-photon calcium imaging^26^. The crucial requirement for successful functional-type accounting is unbiased sampling, over large distances and at cellular granularity. Dense, high-channel-count recording systems are well suited for such applications. Importantly, electrophysiological recordings read out spikes—the transmission protocol between the retina and the brain—rather than somatic calcium fluxes, which are secondary to spike generation.

As a proof of concept, in Fig. 4 we flashed 1122-µm diameter light spots of 1-s duration over the retina while simultaneously recording spikes. Using a high-throughput stream processing pipeline (Supplementary Fig. 5), we sorted and functionally classified the evoked spikes from 1750 neurons in response to the light stimuli. The RGCs were classified according to changes in spiking activities during a period spanning 3-s around the 1-s visual stimuli. Upon satisfying appropriate response characteristics (Methods section), the neurons were assigned one of several classical functional types^31,32^: ON transient, ON sustained, OFF transient, OFF sustained, ON–OFF, and SbC RGCs. Notably, large-scale recordings allowed us to routinely identify and record from the so-called suppression-by-contrast (SbC) RGCs, also known as uniformity detectors. These neurons were first described^33^ in 1967, but seldom studied electrophysiologically^34^, presumably due to rare encounters in low-channel-count recordings. Example spike rasters, for five stimulus repetitions, of each functional class are illustrated in Fig. 4b–g.

The RGC types were homogeneous throughout the field of analyses (Fig. 4a), consistent with known RGC spatial distribution properties^35^. We further analysed the population distributions of the six RGC types (Fig. 4h). Approximately 14% of the neurons were the ON–OFF class, in agreement with anatomical accounting^36^. There was a slight excess of OFF-type neurons comparing to the ON-types, a consequence of some OFF neurons’ smaller dendritic arbor and hence higher density^37^. More sophisticated light stimuli will permit further sub-division of RGC types. Nevertheless, these results illustrate the ability of the processing pipeline and algorithms to analyse the data accurately and automatically. These are important attributes for at-scale analytics.

### Simultaneous microstimulation and recording

Electrical microstimulation has a long application history in neuroscience^38,39^. It provides a method for perturbing the neurons and/or network being studied. Furthermore, neural stimulation at scale may be useful in medical applications, as demonstrated by a 1500-electrode implantable photodiode array, which enabled blind patients to read and navigate^40^. With the exception of a recent design^16^, existing at-scale (>1000 channels) electrophysiological tools have either lacked microstimulation features^11^ or offered limited simultaneously operable stimulation sites (up to approximately three dozen) despite high electrode number^8,19^.

**Fig. 5:**
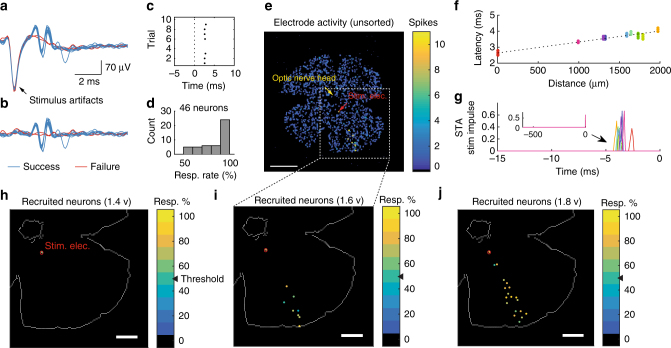
Spatiotemporal effects of microstimulation revealed by large-scale electrophysiology. A grid of 2 × 2 electrodes was used throughout. Besides **h** and **j**, 1.6 v pulses were used. **a** Simultaneous stimulation and recording at one electrode. Superimposed traces from 10 trials. **b** Same data as **a**, after removing the stimulus artifacts. **c** Raster plot for the evoked spikes in **b**. **d** Response rate (10 trials) of 46 RGCs in two retinas. **e** Events detected within 3 ms of stimulus onset. The stimulation site is marked by the red arrow. Aggregated data from 10 repetitions. Scale bar, 1275 µm. **f** Latency of the evoked spikes increased with distance from the stimulation site. Each dot represents a spike (69 in total). The colors denote different neurons. Dotted line is linear fit (*R* ^2^ = 0.9541, *p* < 0.0001, F-test). **g** Time course of spike-triggered average (STA) stimulus for each (color-matched) neuron in **f**. **h**–**j** Spike-sorted activity maps of identified neurons. The number of distant neurons activated by electrical stimulation increased with stimulus strength. The colors indicate response rate over 10 trials. Dotted lines are approximate outline of the retina. Scale bar, 765 µm

Space saving achieved by removing the per-channel antialiasing filters allowed us to implement a stimulator within each recording site. The stimulators are individually programmable. Stimulus artifacts are reduced with two circuit features. First, routing-associated parasitic capacitance is minimized by integrating the stimulation circuit beneath each electrode. The charging and discharging of this capacitance during stimulation manifest as transient artifacts in the recordings. Second, the MOSFET pseudo-resistor in series with the electrode (Supplementary Fig. 2a) is disabled during and immediately after stimulation to quickly restore the first recording transistor’s biasing voltage. Figure 5a illustrates, for ten trials, the electrically evoked spikes of a RGC following a single pulse. These spikes are easily distinguished from the artifacts. Further artifact suppression was achieved by subtracting recordings without neurons from those with neurons (Fig. 5b). The evoked spikes could be detected automatically in these post-processed data using the platform’s stream processing pipeline (Fig. 5c). In this example, the neuron responded in 8 of 10 trials. To assess the reliability of electrical stimulation, we stimulated and calculated the response rate (over 10 trials) of 46 RGCs in two retinas using single 1.6 v pulses (Fig. 5d). More than half of these neurons responded to each trial, while the remaining neurons responded with ≥50% probability.

To ensure that these short-latency spikes were not spontaneous activities, we examined the quiescent firing rate of 22 neurons from a single retina (Supplementary Fig. 6a). The mean firing rate was 2.6 Hz. In contrast, when electrically stimulated, these same neurons spiked with a mean response rate of 74.1% within 3.0 ms of stimulus delivery, over 10 trials (Supplementary Fig. 6b). Therefore, the increased spiking probability following electrical stimulation was unlikely to be of spontaneous origin.

### Loss of focal activation by high-strength microstimulation

An important goal of microstimulation, and indeed for neuronal manipulation in general, is achieving spatiotemporally precise activation. Some studies have found confined activation with single-neuron precision^41,42^, while others observed wide-spread neuronal activation^39,43^. This discrepancy could be a consequence of shortcomings in existing recording tools. First, the inability to record from every neuron, or nearly so, across sufficient area could lead one to incorrectly conclude that activation is spatially confined, because signals from the recruited neurons were not completely accounted for. Second, techniques with limited single-spike sensitivity, such as calcium reporters^44,45^, require large stimuli capable of eliciting multiple spikes to reach detectability threshold. Electric field size increases with stimulus amplitude, influencing a larger neuronal population, giving rise to the alternative, incorrect conclusion of wide-spread neuronal activation, caused by the use of excessive stimuli due to poor spike sensitivity.

A key advantage of cellular-resolution, at-scale electrophysiology is the ability to simultaneously observe activities over the entire retina with single-spike sensitivity. We re-examined the spatial confinement of microstimulation in the retina while stimulating at one location (Fig. 5e). With moderate stimulus strength, we observed a 70% (7 out of 10 trials) response rate from one neuron at the stimulation site (Fig. 5i, red circle). A number of distant neurons were also recruited (i.e., responded in ≥50% of trials). The response latencies increased with distance from the stimulation location (Fig. 5f, g). The axon of RGCs converges at the optic nerve head near the central retina, where they exit the eye. The spatial distribution and response latency of these activated distant neurons were consistent with retrograde axonal stimulation^46,47^, as the axon from these neurons passed in close proximity to the simulation site.

The number of distant neurons recruited by electrical stimulation was strongly dependent on the stimulus strength. Weak stimuli recruited one neuron at the stimulating electrode (i.e., the targeted neuron; Fig. 5h). The number of distant neurons elicited, as well as their response probability, increased as the stimulus strength increased (Fig. 5h–j). Therefore, focal activation depended critically on the stimulus strength. It should be sufficiently powerful to recruit the close proximity, target neuron, but should not be excessive, to avoid stimulating distant neurons with axons passing near the stimulating electrode.

## Discussion

Traditional multichannel electrophysiology is limited in its ability to simultaneously realize low noise, dense and large-scale recordings, due to the need for per-channel antialiasing filters. We presented an acquisition paradigm that does not require these scalability-limiting elements. A platform based on this paradigm allowed us to record spiking activities in the mouse retina across more than 34,000 electrodes with high SNR. In conjunction with the platform’s high-performance computing infrastructure, we were able to sort and functionally classify more than 1700 neurons following light stimulation. Finally, recording at cellular-resolution, across large area and with single-spike sensitivity, allowed us to examine the dynamics of microstimulation in greater spatiotemporal resolution than previously possible.

Our acquisition paradigm is inspired by compressive sensing (CS), based on the central CS notion that, if we know something about the frequency contents of the signal being acquired, it may be possible to recover the signal without sampling at the classical Nyquist rate. In electrophysiology, we know how the aliased thermal noise is manifested in the under-sampled, per-channel data. Conventional CS approach would attempt recovery by some form of iterative, optimization algorithm, which is generally quite slow. In electrophysiology, we could instead exploit the statistical prior of thermal noise for computationally efficient recovery. Thus, while the implementations differ, the general concept is identical. There are further conceptual similarities. CS often uses irregular/random under-sampling to achieve incoherent aliasing. That is, the spectral power of the aliased content is evenly spread out across frequencies constituting the under-sampled data. This is notionally similar to our approach, where the aliased thermal noise is folded down into the first Nyquist zone (Supplementary Fig. 3b), averaging out variations in the original thermal noise power spectra and spectral angles.

Several multichannel electrophysiological systems have recently reached simultaneously recording channel counts in the thousands^11,16,23^ or even up to 16,000 channels^48^, at the expense of noise performance. The noise in these tools are several times higher than traditional systems with at most a few hundred electrodes (≤10 µV rms verses 25–250 µV rms or more). High SNR is critical for spike sorting, where neurons are distinguished on the basis of minute differences in spike waveform. This is particularly relevant for the mammalian nervous system. For example, extracellular signals in the mouse retina generally do not exceed much more than 150 µV peak-to-peak, while reliable spike sorting requires at least 100 µV peak-to-peak signals under optimal SNR conditions^49^. Indeed, current high-channel-count implementations have used higher-than-typical spike detection thresholds^24^ (7.5 SD vs. ~4.0 SD) to avoid misinterpreting noise as spikes; have detected few neurons despite the large number of electrodes^48^ (126 neurons in 16,384 electrodes); or have identified putative events at locations that apparently did not correspond with neurite positions^23^. In general, because of noise, a substantial fraction of neurons may be unobservable when using these systems, potentially diminishing the benefits of high channel count and/or high electrode density. The systems designed by the Litke and Chichilinisky groups^27,50^, and the Roska and Hierlemann groups^8^ have achieved input referred noise as low as 5 µV rms and 2.4 µV rms, respectively. The higher SNR offers several advantages, including improved spike sorting performance and the ability to detect dendritic spikes. However, the superior noise performance limits the simultaneously recording channels to 1024 or less, due to the need for per-channel anti-aliasing filters in these classical multiplexed systems.

Here we demonstrated the multichannel acquisition paradigm in a 65,536-channel ex vivo recording grid, fabricated using commercial CMOS-integrated circuit (IC) technology. The strategy is generalizable to any dense, high-channel count electrophysiological systems, including implantable, long-term in vivo recording tools. In these, multiplexing is important not only for density and channel count scaling, but also to reduce wiring, power consumption and heat dissipation. Indeed, the sampling paradigm will work for any big-data acquisition applications where the spectral and statistical characteristics of the high frequency components, above twice the per-channel observation rate and below the recording channel bandwidth, can be reasonably approximated.

Another advantage of this data acquisition approach is that the signal processing steps (channel separation and aliased noise removal) are all implemented in the digital domain. The throughput of these procedures will improve with technological advancements in electronics, allowing the approach to continue scaling beyond the tens of thousands of simultaneously recording and stimulating channels presented here.

## Methods

### Recording and stimulation architecture

The architecture for our platform is summarized in Fig. 1a. Supplementary Fig. 2a shows the schematic overview for the recording and stimulating circuits. The platform is constructed from a combination of custom IC, circuit-board-level components, synthesized digital logic in field programmable gate arrays (FPGAs) and algorithms running on ×86 CPUs and NVIDIA CUDA processors. The IC (Supplementary Fig. 2b) contained 65,536 front-end elements, divided into 16 blocks of 4096 elements each. Each block is connected to a back-end circuit for additional amplification through a 4096:1 multiplexer. We bandpass filter the outputs from these back-ends to confine spectral content between 50 Hz and 40 MHz with a Sallen-Key filter, implemented on the printed circuit board, then digitize the resulting signals using 12-bit analog-to-digital converters (ADCs). The ADCs’ data streams are captured by a FPGA and transferred to a computer. There are four FPGAs in the system, each handling the outputs of four ADCs. Each front-end element contains programmable registers to enable or disable voltage-based, electrical microstimulation, via the capacitive-coupled HfO_2_ dielectric interface.

The system is powered by a 6 V supply, and uses approximately 24.7 W when in operation. The power consumption is dominated by the four Xilinx FPGAs and, to a lesser extent, the board-level bandpass filters. The IC consumes less than 0.6 % (i.e., ≤148 mW) of the total power budget.

### Electrical stimulation

The electrical stimulation strategy is based on our previous architecture^51,52^. Thirty-two elements are configured at a time during the programming phase, which takes 60 ns. Only elements needing change of stimulation status require programming. Asserting a global digital signal triggers stimulus delivery on all electrodes programmed to do so. User programmable voltage stimuli ranging between 0 to 2.0 volt, are generated by a voltage source on the circuit board (Fig. 1f), fed into the IC, shared by all stimulating electrodes, and capacitive-coupled to the neurons via the electrodes’ HfO_2_ dielectric interface. The MOSFET pseudo-resistor is turned off during stimulation to minimize source resistance. For all electrical stimulation results presented here, we delivered the stimuli with four neighboring electrodes arranged in a 2 × 2 pattern, we found the larger electric field so generated to more consistently elicit spikes in the mouse retina than that from one electrode. The stimulus threshold is defined as the voltage required to elicit electrically evoked responses within 3 ms of stimulus onset in 50% of trials (out of 10), at the 2 × 2 stimulating electrodes.

### Integrated circuit fabrication and post-processing

The top metal layer of the CMOS IC serves as the base material for the sensing electrodes. This is achieved by etching away the foundry-deposited passivation layers (polyimide, silicon nitride and silicon dioxide) by inductively coupled plasma/reactive ion etching (ICP/RIE) using a mixture of SF_6_ and O_2_ plasma. We restrict etching to within the sensing region by protecting all other area with a ~16 µm layer of AZ-4620 photoresist, patterned with standard UV photolithography.

The naturally occurring aluminum oxide on the top metal is stripped by ion milling. Next, we deposit 6 nm of HfO_2_, a high-K dielectric, by atomic layer deposition (ALD) at 150 °C on top of the metal. This serves two purposes. First, it creates a capacitive sensing and stimulation interface; and second, it provides a passivation layer for the underlying aluminum. This HfO_2_ layer provides a capacitance of 5.8 pF over the 14 × 14 µm electrode. The capacitance is ascertained by building test structures (Supplementary Fig. 2c) consisting of a metal-HfO_2_-metal stack on a SiO_2_ substrate, followed by measurements with a semiconductor parameter analyser (Agilent B1500).

A 160-nm-thick film of conductive polymer^10^ is spun over the die surface to reduce the electrode-to-electrolyte interfacial impedance^53^ (between the conformal ALD HfO_2_ layer and saline). This is followed by a 220-nm PMMA A4 (MicroChem) barrier film. The PEDOT:PSS+ PMMA stack is patterned using UV lithography with Shipley S1813 photoresist, followed by O_2_ ICP etching, such that only the electrodes are covered by PEDOT:PSS. Finally, the remaining PMMA is stripped with PG Remover (MicroChem).

Each post-processed die is attached to a custom ball grid array (BGA) with thermally conductive epoxy, wire-bonded, and then encapsulated with medical grade epoxy (OG-116-31, Epoxy Technology, Inc.). In the final step, we attach a polycarbonate ring around the IC using Sylgard 184 (Dow Corning) to serve as the perfusion chamber.

### Sparse sampling and data recovery

Multiplexing causes each channel to be observed at a rate (*f*_visit_), through the multiplexer, considerably lower than the channel bandwidth (*f*_BW_). Unless the content spanning *f*_visit_/2 … *f*_BW_ is removed from the per-channel data, aliasing occurs. The problem of per-channel data recovery is thus two-fold. First, the channel data must be extracted from the ADC data stream (Fig. 1b, step 2); and second, the spectral contribution of contents in *f*_visit_/2 … *f*_BW_ has to be computed (Fig. 1b, step 3) and removed from the channel data (Fig. 1b, step 4).

The first task, per-channel data extraction, is achieved by keeping a history of the scanned channels during recording. In this manner, each sampled value from the ADC can be assigned to the channel from which it originates by examining the history at the corresponding time point.

The goals of the second task are to preserve the spectral contents of neural signal and to prevent aliasing of contents in *f*_visit_/2 … *f*_BW_, for data sampled at only f_visit_ (Supplementary Fig. 3b), with *f*_visit_ ≪ *f*_BW_. We begin by setting the multiplexers’ per-channel visit rate (*f*_visit_) to be sufficiently high, such that the spike bandwidth (300–3k Hz) is entirely encompassed by *f*_visit_/2 and that the range *f*_visit_/2 … *f*_BW_ is dominated by thermal noise. We typically set *f*_visit_ to 10 kHz to achieve these requirements.

Several statistical and spectral characteristics of thermal noise make its aliased image amenable to reconstruction in the frequency domain. This thermal noise, which comes from the electrodes and from the amplifier transistors, is a stationary random process, with a flat spectrum and a Gaussian time-domain amplitude distribution^54^ of zero mean and variance σ^2^. The probability density function for such a process is

$$N(x|\sigma ^2) = \frac{1}{{\sqrt {2\pi \sigma ^2} }}e^{ - \frac{{x^2}}{{2\sigma ^2}}}$$

We can easily determine every channel’s σ^2^ for thermal noise calculation by recording each channel without multiplexer interruption (i.e. conventional sampling) at full system bandwidth, thereby completely specifying the thermal noise characteristics of the channel up to *f*_BW_. With the noise variance *σ* ^2^ and bandwidth *f*_BW_ now known for every recording channel, we computationally construct the thermal noise *n*_*i*_ for each channel *i*.

As the under-sampled thermal noise is folded down into the First Nyquist zone, in the per-channel data, fluctuations in power and spectral angle, from frequency to frequency, are averaged out (Supplementary Fig. 3b). We can compute the power contributed by aliasing using the expected average thermal noise power, multiplied by the number of folded Nyquist zones. Similarly, the spectral angles converge to zero in the aliased version of the thermal noise. We construct vectors in the frequency space to represent the aliased thermal noise, subtracting these from the per-channel data, thereby reversing the effects of aliasing.

The effects of thermal noise aliasing, between *f*_visit_/2 … *f*_BW_, in the per-channel data (Supplementary Fig. 3b) can be readily reproduced by decimating the generated noise *n*_*i*_ to a lower rate, *f*_visit_. We denote this aliased sequence *a*_*i*_:

$$a_i:{\mathrm{decimate}}(n_i,f_{{\mathrm{visit}}})$$

Next we construct another sequence *b*_*i*_, a decimated version of *n*_*i*_ without aliasing. This is accomplished by first low-pass filtering *n*_*i*_ at *f*_visit_/2, followed by decimation to the new rate *f*_visit_:

$$b_i:{\mathrm{decimate}}\left( {{\mathrm{lowpass}}\left( {n_i,f_{\mathrm{visit}}/2} \right),f_{\mathrm{visit}}} \right)$$

The power contributed by the aliased thermal noise at each frequency, for a system with bandwidth *f*_BW_ but sampled at only *f*_visit_, is, therefore, the power difference between the deliberately aliased sequence *a*_*i*_ and the anti-aliased sequence *b*_*i*_:

$${\mathrm{P}}_i = \left| {{\cal F}(a_i)} \right| - \left| {{\cal F}(b_i)} \right|$$

where ${\cal F}$ denotes Fourier transform. By removing the contribution of P_*i*_ in the per-channel data, we avoid aliasing. Because thermal noise is a stochastic process, for any finite-length segment there will be slight fluctuations in power from frequency to frequency, and no two finite-length segments are exactly identical. These uncertainties are minimized with increased length for *n*_*i*_, and by computing P_*i*_ from the averaged power, which converges to the true value as the number of analysed frequencies increases:

$${\mathrm{P}}_i = {\mathrm{mean}}\left( {\left| {{\cal F}\left( {a_i} \right)} \right|} \right) - {\mathrm{mean}}\left( {\left| {{\cal F}\left( {b_i} \right)} \right|} \right)$$

We then construct a set of vectors describing the aliased contents in the frequency domain:

$$\overrightarrow V _\iota = {\mathrm{P}}_ie^{j\,{\mathrm{arg}}\left( {{\cal F}\left( {d_i} \right)} \right)}$$

In the last step, we remove these aliased contents $\overrightarrow {V_i}$ from the per-channel data $d_i$. In doing so, we recover the data $e_i$ without aliasing (Supplementary Fig. 3c):

$$e_i = {\cal F}^{ - 1}({\cal F}\left( {d_i} \right) - \overrightarrow {V_i} )$$

In practice, we perform the thermal noise parameter estimation procedure separately in physiological saline prior to the biological experiments with 50–100 ms recordings at full sampling rate. This can be computed for 16 pixels in parallel, taking advantage of the 16 parallel read-outs on the IC. The noise parameters are saved for each pixel and reused in subsequent biological experiments.

### Data analysis pipeline

The four FPGAs, each collecting digitized data from four ADCs, are connected to a high-performance computer with separate USB3 links (Supplementary Fig. 5), with a combined transfer capacity of approximately 1 GB/s. Low-level drivers and custom libraries store the data to RAID0 hard drives and arbitrate interactions with near real-time processing algorithms written in C++, running on Intel ×86 CPUs (Xeon E5-2623 3 GHz) and NVIDIA GPUs (Quadro K5200).

### Characterization of acquisition paradigm

The perfusion well is filled with PBS at physiological concentration. We generate 1-kHz sine waves from a function generator (AFG3102C, Tektronix) and attenuate the signal amplitude down to 100–200 µV peak-to-peak. The test signals are applied in the PBS bath through a pair of large (hence low impedance) Ag-AgCl electrodes. The recording SNR is measured by applying a 1-kHz sine wave into the saline bath, directly above the test electrode. After bandpass filtering the data between 300 and 3 kHz, we then compare its variance against a similarly bandpass filtered quiescent recording with a grounded bath. We determine the corner frequency of the high-pass filter at each electrode by measuring the amplitude attenuation of a bath-applied sine wave, at different frequencies.

We also compare the performance of our system to that of conventional, low-noise patch clamp recordings. A ~950-kΩ, PBS-filled borosilicate glass pipette, connected to a commercial patch clamp recording setup (MultiClamp 700B, Digidata 1550, pClamp 10, all Molecular Devices), is brought within 50 µm of the test electrode in the 65,536-electrde grid using a micromanipulator (MP-285, Sutter Instruments). The digitally recovered data from the test electrode within the CMOS recording grid is compared against the patch clamp amplifier’s recordings. Both signals are bandpass filtered between 300–3k Hz.

### Mouse retina preparation

WT mice >P40, of either sex, are dark adapted for one hour and deeply anaesthetized with isoflurane in O_2_. Following euthanasia, the eyes are rapidly enucleated under dim red light then placed in oxygenated Ames’ medium (Sigma Aldrich) with 1.9 g/L of NaHCO_3_ and equilibrated with 95% O_2_/5% CO_2_, at room temperature. Under a near-infrared illuminated dissection microscope we hemisect the eyes, remove the anterior chamber, the vitreous and the posterior eye cup, then place the isolated retina in equilibrated Ames’ medium at room temperature, in darkness.

For recordings, an intact retina is flattened by several small incisions around the periphery, transferred onto a transparent dialysis membrane, then placed retinal ganglion cell side down, on top of the 65,536-channel CMOS recording / stimulation grid. A small, custom-made platinum harp, with (Supplementary Fig. 7) or without nylon threads, is placed over the membrane to maintain retina-to-electrode contact. The retina is kept alive by perfusing with equilibrated Ames’ medium, heated to 33–35 °C, at a rate of ~4.5 mL/min. We allow at least 30-minutes recovery in the warm solution before recordings. All experiments are performed in the dark. Visualization of the retina under a fixed-stage upright microscope (Nikon FN1) is achieved with near-infrared illumination (≥850 nm) and an IR-sensitive CCD camera.

Visual stimuli are generated on an OLED display with built-in digital signal processor and memory, then projected onto the retina via custom optics attached to the modified epi-fluorescent light path of a fixed-stage microscope. Visual patterns are triggered via a TTL digital input. The light spot stimuli are 1-s in duration, of 1122 µm diameter, white on black background at 100 % contrast, with intensity of 11.4 × 10^11^ photons/s/cm^2^.

### Data analysis

Eight mouse retinae were used for the biological results presented here. After per-channel data separation and noise removal (Fig. 1b), the data are bandpass filtered between 100 Hz and 3 kHz. Putative spikes are detected by threshold crossing over 4.5 standard deviation of mean. We use an event window of 0.7 ms and 1.0 ms, before and after, the spike peak, respectively. To facilitate spike sorting, whenever an event is detected, we also collect the waveforms from the electrode’s eight adjacent neighbors over the corresponding timeframe. These waveforms are concatenated and saved to a database, mapping from electrode address to event data (peak time, waveform data and recording sweep ID).

We compute the principal components for each waveform by singular value decomposition, then sort the waveforms using the first four scores by expectation maximization (EM) with Gaussian mixture model. We repeat the EM procedure ten times to avoid suboptimal, local-maxima solutions. The repetitions are performed concurrently using parallel CPUs/GPUs to reduce run time. The clustering with the highest fitness metric, namely, maximal between-cluster separation, minimal within-cluster spread, lack of singleton clusters and zero inter-spike interval violation, are deemed the correct/best solution. Conventional EM algorithms require a priori the number of clusters, an impractical requirement for at-scale spike sorting. We implement automatic cluster number detection using the foregoing fitness metric. Specifically, the number is increased incrementally from a minimum of two, up to maximum of 10. The lowest cluster number without inter-spike interval violation and having the highest, or equal highest, fitness metric is used. These procedures are implemented on parallel hardware to reduce run time. All sorted spikes are saved to a database, mapping from electrode address to a list of waveforms, their associated spike time, sweep ID and cluster assignment.

To classify RGCs into functional types^31,32^, we flash a 1-s light spot over the region of interest in the retina. The recording duration is three seconds, for 1-s pre-stimulus and 1-s post-stimulus periods. We divide the 3-s recording interval into six equal segments of 500 ms each, numbered 1–6. ON-type neurons spike predominantly in segments 3 and 4, while OFF-type neurons spike predominantly in segments 5 and 6. Neurons with high segment 3 or segment 5 rates relative to segments 4 and 6 are designated as “Transient”, otherwise they are designated as “Sustained.” ON–OFF RGCs are distinguished by simultaneously strong responses in segments 3 and 5, and low baseline spiking rates. SbC RGCs are distinguished by their high baseline rate, with little or no spikes in segments 3 and 5. Units with ambiguous spiking profile, without the foregoing characteristics, are not assigned a functional class. Units with low spike rate (≤2 Hz) during the 3-s recording period are also not functionally classified, because the low spike counts preclude accurate classification. The pseudocode for the functional classification procedure can be found in Supplementary Note 1.

In Fig. 5, the spike-triggered average (STA) stimulus is defined as the average stimulus preceding a spike from a neuron. Specifically, it is the sum of stimuli (voltage impulses through the HfO_2_ dielectric) that preceded each spike, divided by the number of spikes.

To create the spike latency verses distance plot (Fig. 5f), we begin by collapsing, for each electrode, the spike-sorted events over 10 stimulus repetitions into a time-invariant plot. From this plot, at each electrode, we select all spike-sorted unit(s), with at least 5 events within 5 ms following stimulus delivery (i.e., 5 successes out of 10 trials). The spike time of these units are then plotted as a function of distance from the stimulation site.

### Data availability

The data sets generated during and/or analysed during the current study are available from the corresponding author on reasonable request.

## Electronic supplementary material

Supplementary Information
