# Addendum: A very large-scale microelectrode array for cellular-resolution electrophysiology

**DOI:** 10.1038/s41467-018-06969-6

**Published:** 2018-10-24

**Authors:** David Tsai, Daniel Sawyer, Adrian Bradd, Rafael Yuste, Kenneth L. Shepard

**Affiliations:** 1 Department of Electrical Engineering, Columbia University, New York, NY 10027 USA; 2 Departments of Biological Sciences and Neuroscience, Columbia University, New York, NY 10027 USA; 3 Departments of Electrical and Biomedical Engineering, Columbia University, New York, NY 10027 USA

**Keywords:** Electrophysiology, Retina, Techniques and instrumentation

Addendum to: *Nature Communications*; 10.1038/s41467-017-02009-x; published online 27 November 2017

The noise-reduction algorithm as presented in the Methods section “sparse sampling and data recovery”^1^ seeks to estimate and reduce the spectral contribution of aliased thermal noise, to improve the signal-to-noise ratio (SNR) for highly multiplexed neural recording systems, where implementing adequate antialiasing filters is a challenge. Unfortunately, because of errors described in our corrections^3^ to a companion paper describing the details of these signal processing algorithms^2^, the techniques employed do not improve the SNR of high-density acquisition systems limited by noise aliasing beyond what is achievable with conventional band-pass filters.

Here we summarize the incorrect statements regarding the effects of thermal noise aliasing, incorrect assertions in the paper on our ability to remove aliased noise, the consequence of these errors on the general applicability of the noise-reduction algorithm, and our revised recommendations on processing under-sampled spike (action potential) recordings. The discussion of the trade-off between area and noise for multiplexed acquisition systems, our reports on the recording array hardware and software, and the biological demonstration of the system’s recording and electrical stimulation performance remain valid. We will also show here that while the algorithm does improve SNR for action potential recordings from highly multiplexed acquisition systems with incomplete antialiasing, as a result of these errors, the signal processing efforts employed deliver little utility over a linear band-pass filter in most cases.

There were two errors made in the Methods section “Sparse sampling and data recovery” and the Results section “Compressed sensing-inspired electrophysiology” that affect the treatment of both the amplitude and phase of noise estimates. A thorough treatment of these errors can be found in ref. ^3^ and are summarized below. These have implications on the efficacy of the noise-reduction algorithm and the de-noising claims made in the paper.

The first of these errors was the assumption that the under-sampled thermal noise *averages* in the first Nyquist zone during aliasing, when it in fact sums. This error affects the amplitude estimates of the aliased noise.

The second consequential error affects the phase estimation of aliased noise. In the Methods section, it is stated that aliasing causes a convergence to zero phase for thermal noise folded into the first Nyquist zone, which is incorrect. Furthermore, as pointed out previously, aliasing sums, rather than averages, the down-folded complex terms.

As a consequence of the foregoing errors, the noise-reduction algorithm, therefore, does not mimic the uniform spectral spreading of aliased contents by compressive sensing, through randomized sampling, as stated in the Results and Discussion sections.

Our follow-up analysis in ref. ^3^ shows that the algorithm (SRMA) favors signals with a narrow frequency spectrum, such as, in the limiting case, the sinusoids we used in ref. ^2^. As the signal bandwidth broadens, one would typically expect a decrease in performance. Importantly, while the algorithm does improve the SNR of under-sampled spike recordings, it does not perform better than a band-pass filter^3^.

To assess how aliasing and signal processing (comparing non-aliased recording with band-pass filtering of aliased recording and with SRMA-processing of aliased recording) impact biological analyses, we used recordings from a 60-channel Multi Channel Systems (MCS) multi-electrode array, a widely used setup for retinal electrophysiology without signal aliasing. The electrode array configuration is comparable to our setup, with 10-µm diameter electrodes of 30 µm pitch. Each recording channel has 3 kHz bandwidth. Similar to our test configuration in ref. ^3^, we added noise penalties to account for the aliased thermal noise. This scheme allows us to assess how aliasing and processing algorithms impact spike analyses, with thermal noise aliasing being the only variable, while simultaneously providing a benchmark for “correct” results. For these set of experiments, the retinal neurons were stimulated with a 1 s light spot, as described in the original paper^1^. The spike detection and sorting strategy are also as described previously.

Figure 1a shows the raster plot for a single recording. Non-aliased recording (Conv) through the MCS system registered 83 spikes. Thermal noise aliasing degrades SNR^3^, thus reduced the number of detectable events (spikes) considerably, regardless of post-processing strategies (Ali + BP and Ali + SRMA). These lost events are quantified as false negatives in Fig. 1b across five repetitions of the 1 s, light-flash. On rare occasions, we also observed false positive events (Fig. 1c; five repetitions).

**Fig. 1:**
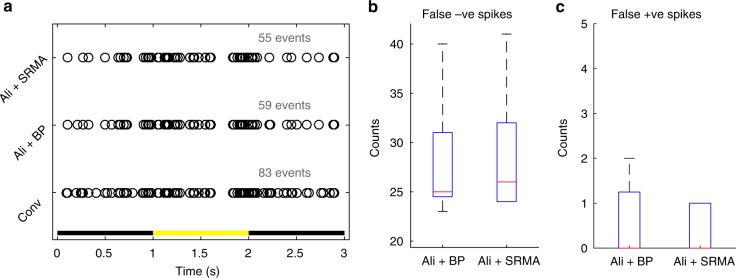
Impacts of aliasing on spike detection performance. **a** The retina was stimulated with a 1 s light flash. Comparing to non-aliased recording (Conv), aliased recordings (Ali), regardless of processing strategy (bandpass; BP; 300–3k Hz or SRMA) registered less events. Summary of false positive (**b**) and false negative (**c**) events across five repetitions of the 1 s light flash

We also spike sorted the recordings. Spike sorting accuracy is also impacted by thermal noise aliasing (Fig. 2). While the rasters for Cells 1–5 are qualitatively similar across the three conditions, some inaccuracies are evident. The spikes from Cell 6, which had small-amplitude action potentials, were almost entirely lost in the aliased recordings, which is true for both BP- and SRMA-processed traces.

**Fig. 2:**
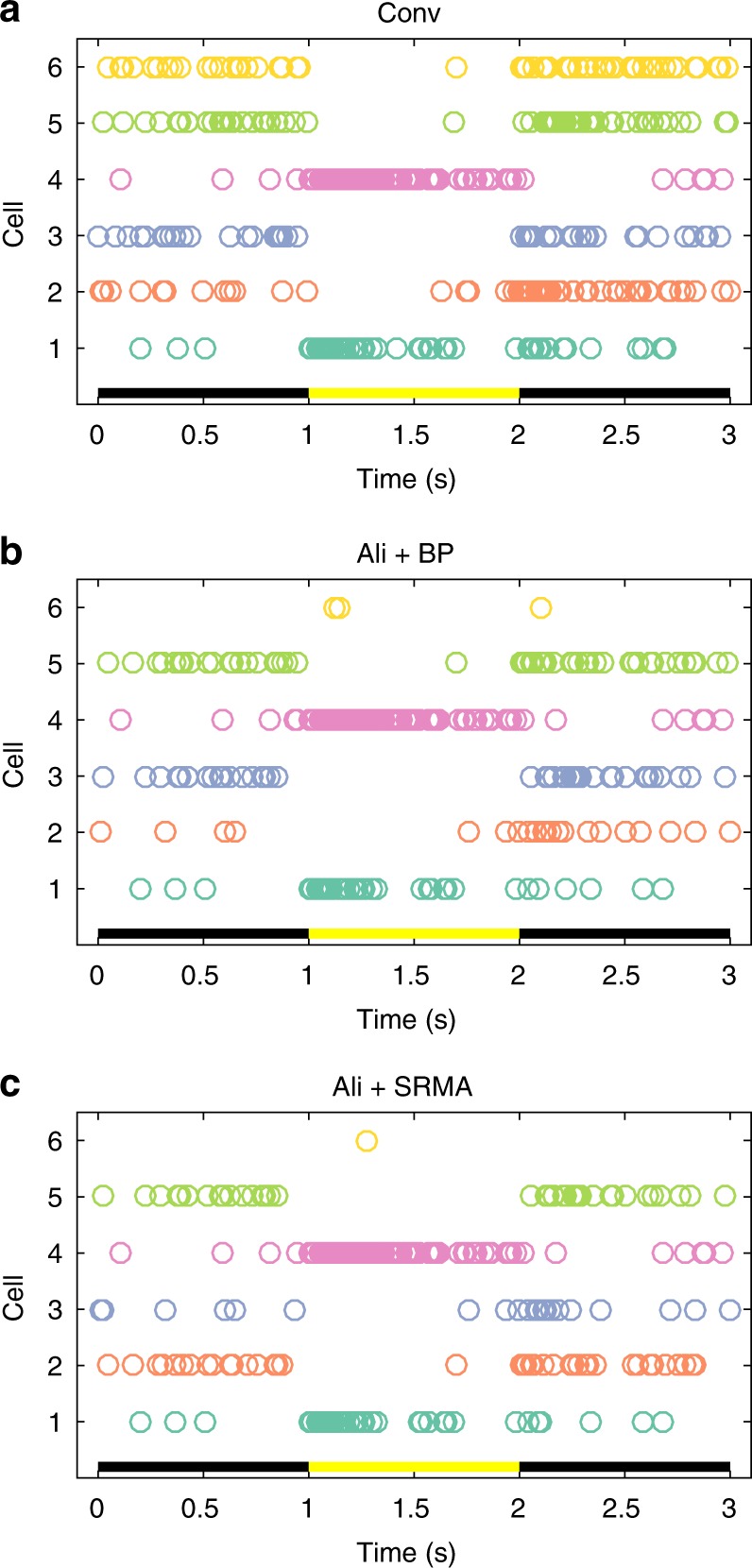
Impacts of aliasing on spike sorting performance. **a** Conventional, non-aliased recordings. **b** Aliased recording with band-pass filtering. **c** Aliased recording with SRMA processing

In summary, we show that the noise-reduction algorithm improves the SNR for under-sampled spike recordings, just as it does for sinusoids, which we demonstrated in ref. ^1^. However, as implemented in ref.^1^, the algorithm does not improve the SNR of under-sampled spike recordings beyond those achieved by a band-pass filter in a statistically significant manner. The residual SNR degradation due to thermal noise aliasing reduced the number of detectable events. Consequently, this impacts spike sorting accuracy, particularly for neurons with small-amplitude action potentials. Contrary to the proposition made in our paper, in the absence of per-channel filters, the SNR of high-density acquisition systems will be limited by noise aliasing, regardless of the post-processing method employed, whether this be SRMA or conventional band-pass filters.

## References

[1] Tsai D, Sawyer D, Bradd A, Yuste R, Shepard KL (2017). A very large-scale microelectrode array for cellular-resolution electrophysiology. Nat Commun..

[2] Tsai D, Yuste R, Shepard KL (2017). Statistically reconstructed multiplexing for very dense, high-channel-count acquisition systems. IEEE Trans. Biomed. Circuits Syst..

[3] Tsai D, Yuste R, Shepard KL (2018). Correction to ''Statistically reconstructed multiplexing for very dense, high-channel-count acquisition systems''.. IEEE Trans. Biomed. Circuits Syst..

