# Supplementary material for: A very large-scale microelectrode array for cellular-resolution electrophysiology
Source: Nat Commun. 2017 Nov 27;8:1802. doi: 10.1038/s41467-017-02009-x (PMC5702607; doi:10.1038/s41467-017-02009-x)
Supplement: Supplementary file 1 — Supplementary Information [file 41467_2017_2009_MOESM1_ESM.pdf]

Supplementary Figures

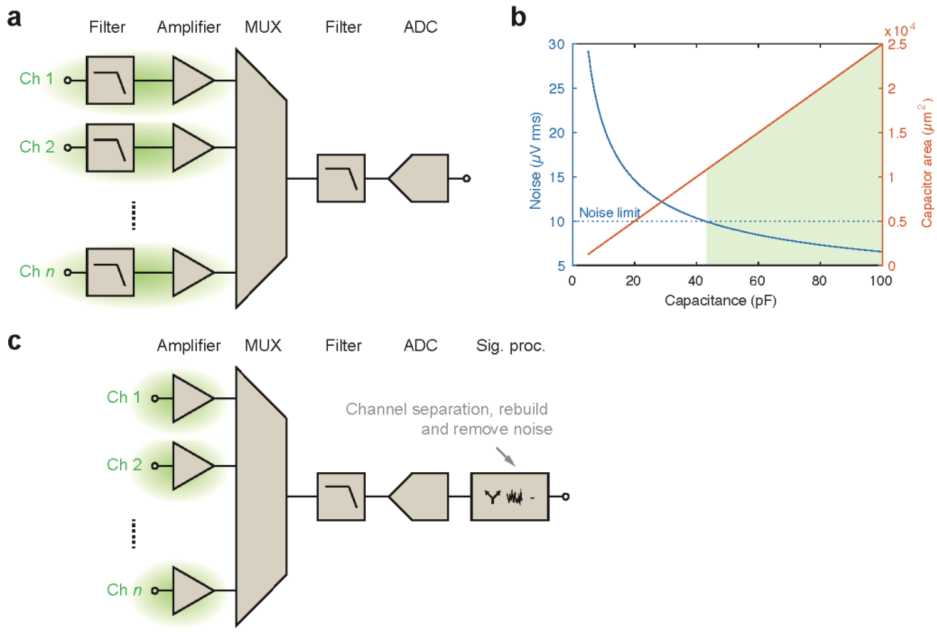

Supplementary Figure 1

Comparison of traditional time-division multiplexed systems and our acquisition paradigm. (a) Conventional multiplexing contains a low-pass filter, at each channel front-end, to prevent aliasing. In the most compact form, these filters consist of a resistor R and a capacitor C. (b) The noise of this RC network is resistance-insensitive[1], having mean-squared voltage kT/C, where k, T, and C are the Boltzmann constant, temperature and capacitance, respectively. More than 40 pF is required to achieve 10 μV rms noise when operating at 35 ºC. Using metal-insulator-metal (MIM) capacitors in high-quality, commercial-grade microelectronic processes[2], each capacitor would occupy > 10,000 μm^2, an area > 20 times the typical mammalian soma. The green-shaded region represents the design space for adequate noise performance of approximately ≤ 10 μV rms. By using varactors, having approximately twice the capacitance density of MIM capacitors, the device-to-neuron area ratio would be 10:1. However, varactors have voltage-dependent capacitance, which could cause signal distortion. (c) Our sampling paradigm, based on sparse sampling, reconstructs and removes the spectral contribution of the aliased thermal noise, obviating the need for per-channel antialiasing.

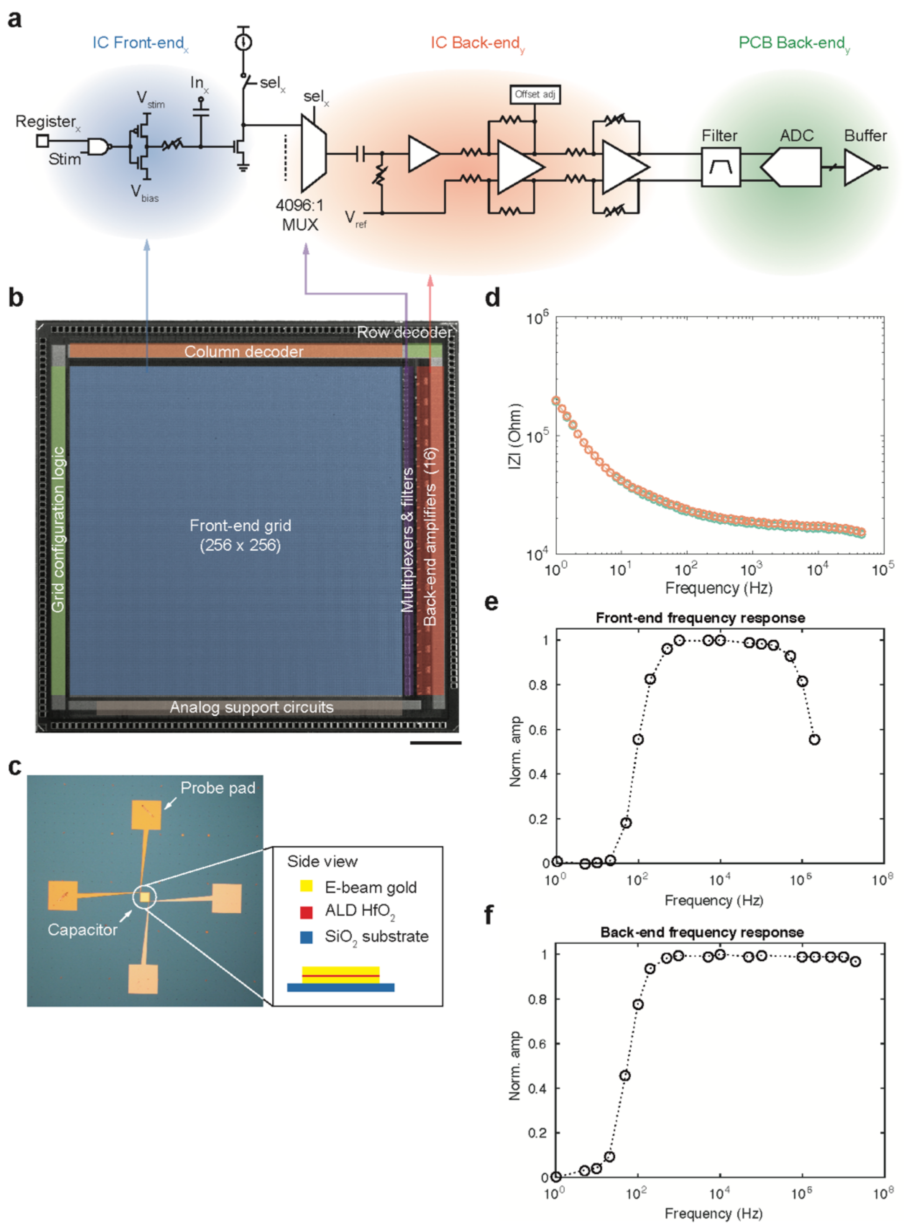

Supplementary Figure 2

Circuit architecture and evaluation of the 65,536-electrode recording and stimulation grid. (a) Overview of the CMOS integrated circuit (IC) and analog-to-digital converter (ADC) on the printed-circuit board (PCB). The 65,536-electrode grid is divided into 16 equal regions, each containing 4096 front-end elements and one shared back-end amplification chain, through a 4096:1 multiplexer. The analog signal from the back-end is subsequently band-pass filtered by a Sallen-Key filter and captured by an ADC. (b) Floor plan of the 65,536-electrode CMOS recording and stimulation grid. Scale bar, 1 mm. (c) Test

structure for measuring the capacitance of the HfO_2 dielectric layer above each electrode. (d) Impedance of two PEDOT:PSS electrodes, measured in phosphate buffered saline. (e) Frequency response of the IC front-end (65,536 elements). (f) Frequency response of the shared back-end (16 elements).

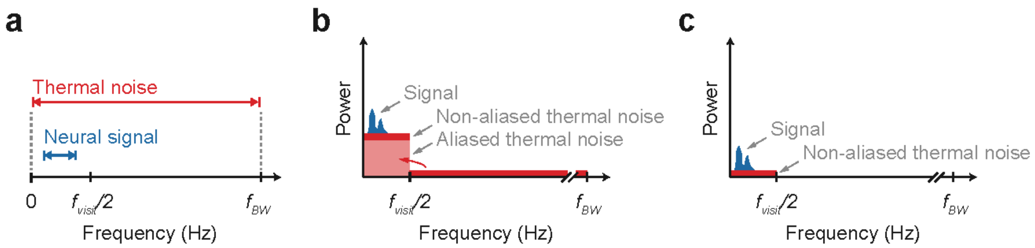

Supplementary Figure 3

Frequency domain illustration of our sparse sampling strategy. (a) We choose the per-channel visit rate by the multiplexer (f_{visit}) and the system's bandwidth (f_{BW}) so that the neural signal bandwidth is preserved, while permitting accurate reconstruction and removal of the spectral contribution from the under-sampled thermal noise. (b) By removing the spectral contribution of the aliased thermal noise, having original, non-aliased, bandwidth f_{BW}, from the data observed at only f_{visit} (pink), we can recover the signal (blue) without aliasing. This is made possible by the known statistical and bandwidth characteristics of thermal noise in our system, and by the averaging properties conferred by thermal noise aliasing. (c) The result is improved SNR, due to the reduced spectral contribution by noise.

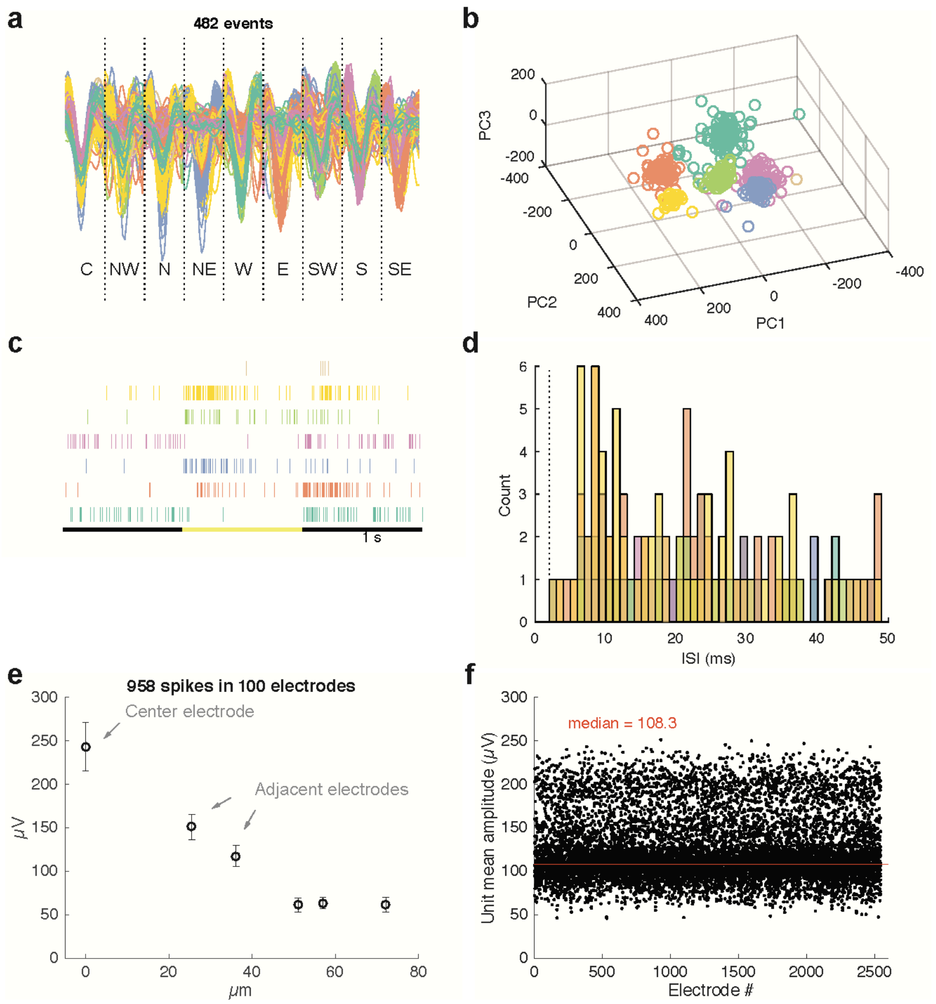

Supplementary Figure 4

Improving spike sorting accuracy through high-density recordings. (a) For each electrode being spike sorted, we concatenated the time-matched waveforms from eight adjacent neighbours to those of the center (target) electrode. The waveforms are color-coded by cluster identity. (b) Spikes in (a) sorted and plotted in the first three principal component space. (c) Raster plot of the spikes in (a). (d) No cluster contained inter-spike intervals ≤ 2.5 ms, which would be indicative of poor sorting. (e) Spike amplitude (mean ± SEM, 100 electrodes) verses distance. For each electrodes analysed (center electrode), the neuron with the largest spike was first identified. We then examined the amplitude of the spikes from this neuron when observed from more distant electrodes. (f) Spike amplitude distribution for all identified units at electrodes encompassed by a 1-s light flash. Each dot represents a unit.

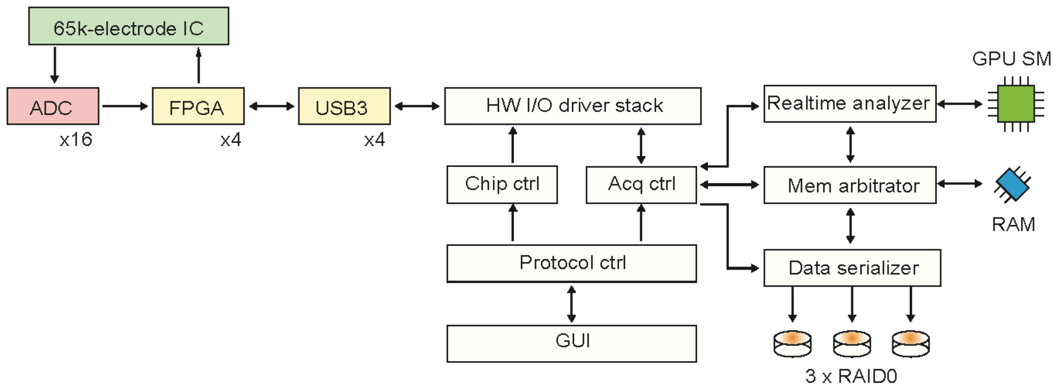

Supplementary Figure 5

Overview of the stream processing pipeline. Data from the 65,536-electrode CMOS IC recording grid are sampled by the ADCs. The output of each ADC is collected by a FPGA and transferred to a high-performance computer, containing multiple Intel CPUs, NVIDIA GPUs and solid-state hard drives in RAID0 configuration. Custom software and libraries running on the computer save the data for later retrieval and for analysis.

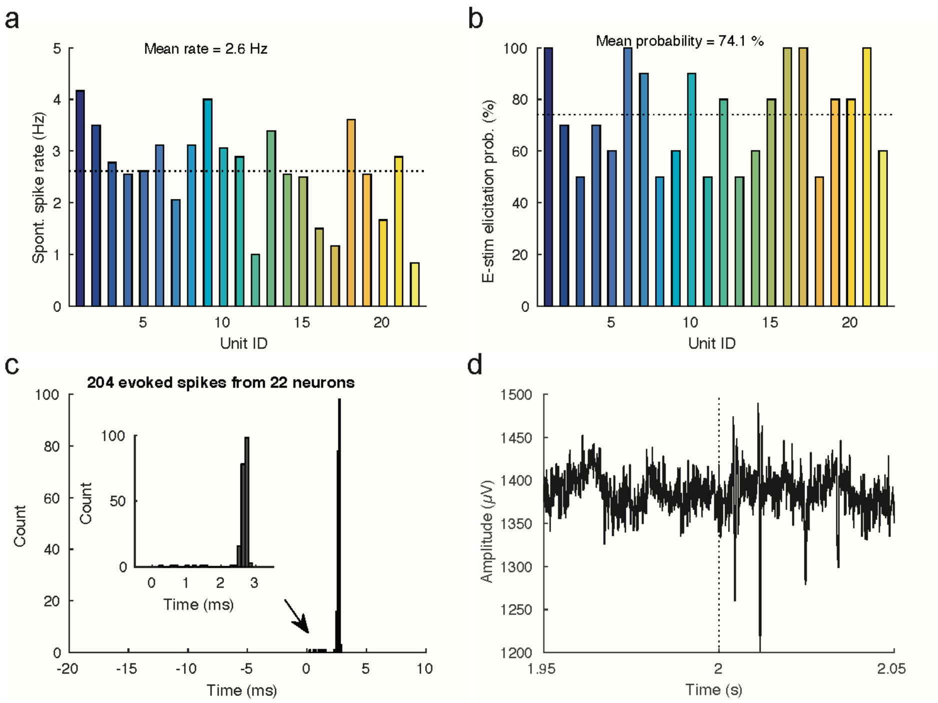

Supplementary Figure 6

Spontaneous spike rate and electrically evoked response rate for 22 RGCs from the same retina. (a) Spontaneous spike rate, over 39 s. (b) Response rates, over ten trials, to a 1.6 V extracellular pulse for the same neurons in (a). (c) Latencies of electrically evoked spikes. Inset: expanded view of the time spanning -0.5 – 3.5 ms after stimulation. (d) Minimal light stimulation artifact. The stimulus was a 1-mm white spot centered over the electrode, beginning at time t = 2 s. The trace has not been filtered.

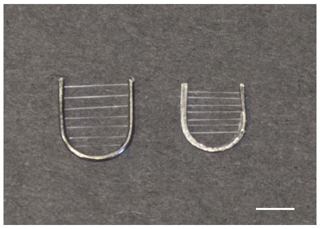

Supplementary Figure 7

Examples of custom-made harps with nylon threads used in some of our experiments. Scale bar, 5.1 mm.

Supplementary Notes 1

The following pseudocode illustrates how we classified each sorted unit into one of the following functional classes: ON T, ON S, OFF T, OFF S, ON-OFF, SbC and ambiguous.

```
% segment spike times into 6 bins of 0.5 s each
clustBins = segment(spikeTimes, 6, 0.5)

% rank bins in descending size
rBins = sort(clustBins)

% average rate within 500ms of light change
transEdgeAvg = (clustBins(3)+clustBins(5)) / 2
% spike rate before stim
preRate      = (clustBins(1) + clustBins(2)) / (duration/6*2) / trials
% spike rate during 1-s stim
stiRate      = (clustBins(3) + clustBins(4)) / (duration/6*2) / trials
% spike rate 500ms after light change
stiTRate     = clustBins(4) / (duration/6) / trials
% spike rate 1s after stim
endRate      = (clustBins(5) + clustBins(6)) / (duration/6*2) / trials
% spike rate 500ms after light off
endTRate     = clustBins(6) / (duration/6) / trials

% classify neuron
if stiRate <= 2 && endRate <= 2
    % indistinguishable type
elseif (rBins(5)==3 || rBins(5)==5) && (rBins(6)==3 || rBins(6)==5) &&
        abs((stiTRate+endTRate)/2 - preRate) / preRate <= 0.25
    % SbC type
elseif (rBins(1)==3 || rBins(1)==5) && (rBins(2)==3 || rBins(2)==5) && ...
        abs(clustBins(3)-transEdgeAvg) <= transEdgeAvg*0.3 && ...
        abs(clustBins(5)-transEdgeAvg) <= transEdgeAvg*0.3
    % on-off type
elseif clustBins(3) + clustBins(4) > clustBins(5) + clustBins(6)
    if clustBins(3) > clustBins(4) * 2.5
        % on transient type
    else
        % on sustained type
    end
elseif clustBins(5) + clustBins(6) > clustBins(3) + clustBins(4)
    if preRate <= 2
        % off transient type
    else
        % off sustained type
    end
end
```

Supplementary References

1 Razavi, B. *Design of analog CMOS integrated circuits*. (McGraw-Hill, 2000).

2 IBM Microelectronics Division. *CMOS7RF (CMRF7SF) Design Manual*. (IBM Corp., 2008).
